# The opto-locomotor reflex as a tool to measure sensitivity to moving random dot patterns in mice

**DOI:** 10.1038/s41598-018-25844-4

**Published:** 2018-05-16

**Authors:** L. A. M. H. Kirkels, W. Zhang, M. N. Havenith, P. Tiesinga, J. Glennon, R. J. A. van Wezel, J. Duijnhouwer

**Affiliations:** 1 Department of Biophysics, Donders Institute, Radboud University, Nijmegen, The Netherlands; 2 Department of Neuroinformatics, Donders Institute, Radboud University, Nijmegen, The Netherlands; 3 Department of Cognitive Neuroscience, Donders Institute, RadboudUMC, Nijmegen, The Netherlands; 4 Biomedical Signals and Systems, MIRA, Twente University, Enschede, The Netherlands; 5 Center for Molecular and Behavioral Neuroscience, Rutgers University, Newark, USA

## Abstract

We designed a method to quantify mice visual function by measuring reflexive opto-locomotor responses. Mice were placed on a Styrofoam ball at the center of a large dome on the inside of which we projected moving random dot patterns. Because we fixed the heads of the mice in space and the ball was floating on pressurized air, locomotion of the mice was translated to rotation of the ball, which we registered. Sudden onsets of rightward or leftward moving patterns caused the mice to reflexively change their running direction. We quantified the opto-locomotor responses to different pattern speeds, luminance contrasts, and dot sizes. We show that the method is fast and reliable and the magnitude of the reflex is stable within sessions. We conclude that this opto-locomotor reflex method is suitable to quantify visual function in mice.

## Introduction

In recent years, mice have become an important animal model for studies on visual processing. Although mice rely much less on vision than primates, their visual system largely resembles that of higher mammals^1–4^. Moreover, mice offer unique opportunities to study the functional circuitry of vision. For example, through the invention of genetically encoded calcium current and voltage indicators it is possible to record in parallel the activity of hundreds of neurons in cortex in the behaving mouse^5–10^. The activity of large groups of neurons can be altered by applying optogenetics^11–14^ or activated by designer drugs^15,16^.

These neurophysiological techniques offer the opportunity to directly relate neuronal function in retina and visual cortex to visually driven behaviour^2,17–20^. For this purpose, suitable tests of visual function are needed. Over the years many behavioural tests for mouse visual function have been developed^21–23^. Visual function in mice is traditionally tested with simple behavioural paradigms based on reflexes, such as the eye blink reflex, the pupil light response^21,24^, light-dark transition test^25^ and optokinetic reflexive responses of the eye or the head to large moving patterns^26–29^. These reflexes are thought to rely on the retino-tectal pathway, which is strongly developed in the mouse^30–32^. Higher perceptual facilities (pattern, depth, orientation) are thought to rely more on retino-cortical pathways. Examples of tests that probe these are the visual cliff test^33^ and modified Morris water maze tests^34–37^.

Many recent studies of mouse visual perception rely on fixating the head in space. This has the advantage of increased control over the visual stimulation and it provides the stability that is typically required in neural techniques such as two-photon calcium imaging^20,38,39^ and single unit or patch-clamp recording^40,41^. Perceptual tasks that head-fixed mice perform concurrently with these neurophysiological assays typically involve licking responses or lever presses to visual stimuli according to specific, trained rules of varying complexity^21,40,42–47^.

The purpose of this study is to test a method we developed that leverages the opto-locomotor reflex (OLR) in a head-fixed paradigm. This reflex effectively stabilises whole-scene motion by cancelling it with a body orienting movement of the same magnitude^46–50^. In this study, we fixed mice with their heads over the center of a spherical, 2D treadmill. All mice spontaneously started running forward. The treadmill was located at the center of a 112 cm dome on the inside of which we projected moving random dot patterns. The presentation of rightward or leftward moving patterns caused the mice to reflexively change their running direction. We quantified the OLR by recording the rotation of the treadmill along the vertical axis. Because this measure is based on reflexive behaviour, it should not require lengthy training like other perceptual assays might.

Our first aim is to assess the sensitivity of the OLR to the stimulus parameters speed, contrast, and dot size. Fixating the heads of the mice provides increased control over the visual stimulation and maximises stability. However, it also eliminates the compensating effect of the OLR on the visual input, as the head remains stable in space while the OLR is transferred to the treadmill. Our second aim is to establish whether or not this causes the reflex to extinguish over time.

## Results

**Figure 1:**
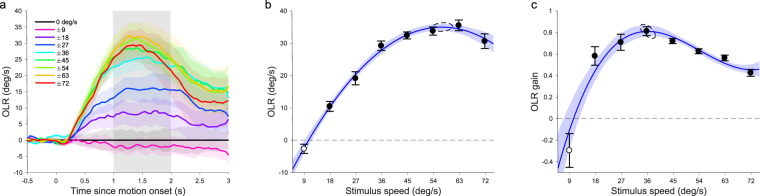
Effect of speed on OLR in mice. (**a**) The mean opto-locomotor response (OLR) over time of all animals (n = 6) to a range of stimulus speeds. (**b**) Mean OLR between 1 and 2 seconds after motion onset (shaded area in **a**) as a function of stimulus speed. (**c**) OLR gain (OLR divided by the stimulus-speed that evoked it) plotted against stimulus speed. Dots had a 1.4 degrees radius and a contrast of 0.68. Shaded bounds in a and error bars in b and c represent SEM. Shaded bounds in b and c represent 95%-CI of the bootstrapped fits. Dashed ellipses represent 95%-CI of the peak estimates of those fits. Open markers in b and c indicate no significant difference from 0 (t-test, p > 0.05).

After one week of habituation to the set-up, the experimental measurements began. In the first experiment the dots moved left or right at one of 9 speeds between 0 and 72 deg/s. These conditions were presented in a randomly interleaved block design. The contrast was held constant at 0.68 and the dot radius was 1.4 degrees. Figure 1a shows the 6-mouse mean, baseline-corrected, OLR-traces over time. Baseline-corrected OLR-traces were calculated, per mouse, by subtracting the zero-speed curve from all its other curves and taking the mean of the pairs of curves observed for equal magnitude rightward and leftward motion, after multiplying the latter by minus one.

When the dots were moving in one direction the animals reacted, on average, by attempting to turn in the same direction, making the treadmill turn (yaw) the opposite way. The peak amplitude of the OLR-traces varied across individual animals, with an almost threefold difference in reflex amplitude between the weakest and the strongest responder. This is reflected in the width of the shaded error bands which represent standard error (Fig. 1a).

To summarise the OLR-traces with a single number, we defined OLR as the mean baseline-corrected yaw (in deg/s) observed between 1 and 2 seconds after motion onset. Figure 1b shows the dependence of the 6-mouse mean OLR on stimulus speed. Higher stimulus speeds evoked greater OLR, but the rise in the OLR attenuated above 36 deg/s and dipped slightly at the highest speed (72 deg/s). To prevent mice with the largest responses from dominating the means, we z-scored the individual OLR-vs-speed curves and scaled them back by multiplication with the standard deviation (SD) of the population and addition of the population mean. This normalization step was performed in all the analyses described in this study.

We used Matlab’s fit function to optimize a quadratic polynomial to the mean OLRs in the linear least square sense, linearly weighted by the inverse of the standard errors (R^2^ = 0.99). F-tests revealed that this gave a significantly better fit than a line (F(1,5) = 248.7, p < 10^−4^) and that a cubic polynomial was excessive (F(1,4) = 0.005, p = 0.95). To determine the speed that evoked the strongest OLR with 95%-confidence intervals (CI) we used a resampling method. We fitted quadratic polynomials to 200 random samples (with replacement) from the 48 data points (6 mice × 8 speeds). On average, they peaked at a stimulus speed of 57.2 deg/s (SD 1.47) reaching an OLR of 35.0 deg/s (SD 0.55).

To gain insight in how well the mice followed the stimulus motion and would have compensated for it had they not been head-fixed, we expressed the response as an OLR gain. That is, the OLR divided by the speed of the stimulus used to evoke it (Fig. 1c). This relation was best characterized by a cubic polynomial (R^2^ = 0.92; quadratic vs cubic: F(1,4) = 10.96, p = 0.029; cubic vs quartic: F(1,3) = 9.68, p = 0.053). Interpolation of this curve revealed that the mice tracked the stimulus motion most closely when it moved at 36.4 deg/s (SD 1.07) by turning the ball at 29.5 deg/s in the opposite direction. In other words, they reached a gain of 0.81 (SD 0.02).

**Figure 2:**
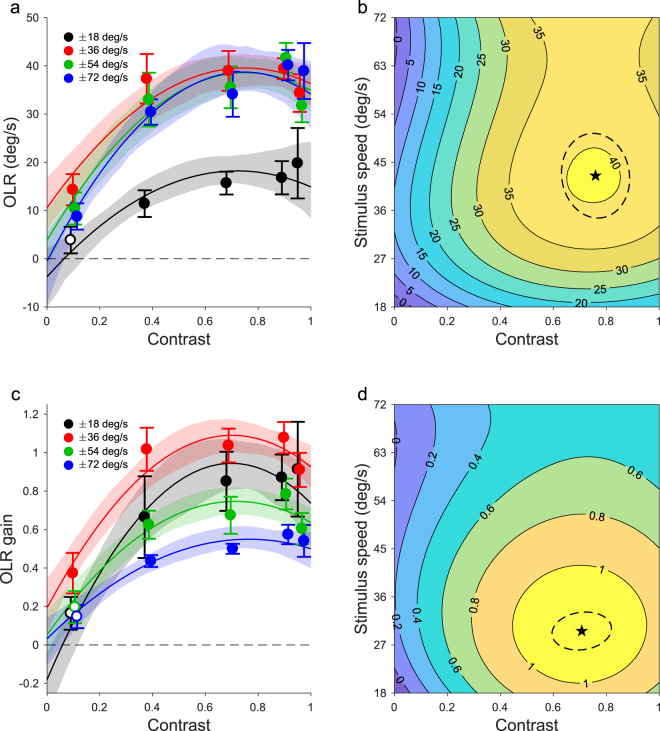
Effect of contrast on OLR of mice. (**a**) Mean OLR for different stimulus speeds plotted as a function of contrast. Dots had a 1.4 degree radius. Error bars represent SEM (n = 6). We bootstrapped the data and fitted OLR vs speed and dot size surfaces. Shaded areas represent the 95%-CI of the cross-sections of these surfaces at the speeds used. (**b**) A contour plot of the mean bootstrapped surface. The asterisk indicates the optimal dot size and stimulus speed with 95%-CI (dashed ellipse). (**c**,**d**) As (**a**,**b**) but for OLR gain. Open data markers in a and c indicate no significant difference from 0 (t-test, p > 0.05). For clarity, all markers in a and c except the red ones are shifted sideways a little.

In the second experiment, we manipulated the Michelson contrast of the dots (0.09, 0.37, 0.68, 0.89, 0.95) and varied their speed (18, 36, 54, 72 deg/s). The dot radius was again held constant at 1.4 degrees. Figure 2a shows mean OLR (n = 6) as a function of the contrast of the dots plotted for each stimulus speed in different colors. As expected, OLR increased with increasing contrast. However, OLR dipped slightly at the highest contrasts used (0.95).

To quantify these results, we fitted the mean OLRs with a polynomial surface function of contrast and speed, linearly weighted by the inverse standard errors. We found that a 9-parameter polynomial that was quadratic for contrast and cubic for speed (poly23 for short) fitted the data well (R^2^ = 0.96). F-tests revealed that this model was significantly better than the simpler, 6-parameter biquadratic alternative (poly22 vs poly23: F(3,11) = 4.70, p = 0.024) and that the addition of more parameters was not warranted (poly23 vs poly33: F(1,10) = 0.70, p = 0.42).

The position of the peak of the surface corresponds to the contrast and speed that evoked the strongest OLR (Fig. 2b). We used resampling to obtain these values with 95%-CI. The mean of the 200 surfaces fit to the resampled data is shown as a contour plot in Fig. 2b. The optimal contrast was 0.76 (SD 0.05) and the optimal speed 42.5. deg/s (SD 3.23).

We analyzed in a similar fashion the effects of speed and contrast on the OLR gain. These data (Fig. 2c) were also best described by poly23 (poly22 vs poly23: F(3,11) = 7.08, p = 0.006; poly23 vs poly33: F(1,10) = 2.46, p = 0.15). We found that the highest OLR gain (1.13, SD 0.05) occurred at a contrast of 0.71 (SD 0.05) and a speed of 29.6 deg/s (SD 1.4) as indicated in Fig. 2d.

**Figure 3:**
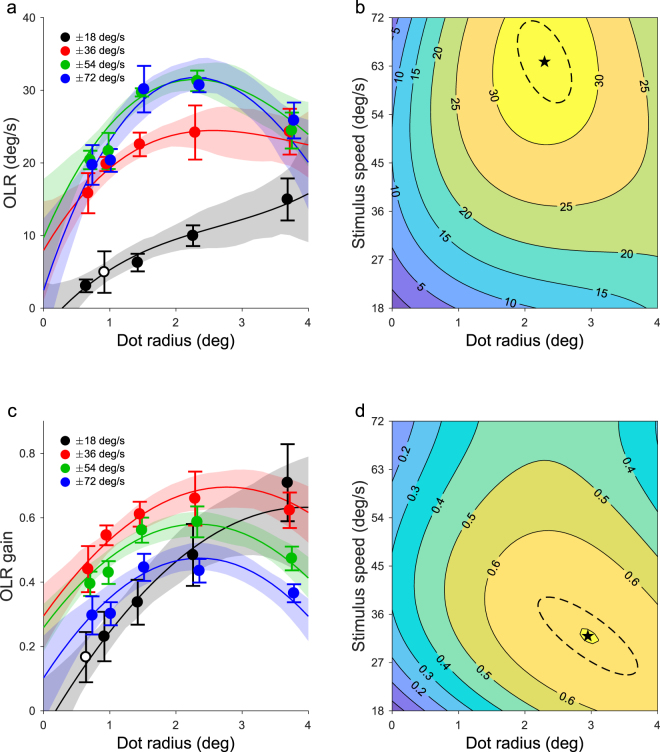
Effect of dot size on OLR of mice. Same as Fig. 2 but for dot size instead of contrast. Contrast was held constant at 0.68.

In Experiment 3, we tested whether there was an effect of dot size on OLR. Figure 3a shows the mean OLR (n = 6) to stimuli with one of six dot radii (0.6, 0.9, 1.4, 2.3, 3.7 deg) and one of four speeds (18, 36, 54, 72 deg/s). We found that OLR is almost linearly related to dot size at 18 deg/s, the lowest speed used. For the higher speeds, we found an inverted U-shape relation between OLR and dot size with the optimum around 2 degrees radius.

These data were best described with a 9-parameter polynomial that was cubic for dot size and quadratric for speed (poly32, R^2^ = 0.99; poly22 vs poly32: F(3,11) = 7.09, p = 0.006; poly32 vs poly33: F(1,10) = 0.78, p = 0.40). The strongest OLR was evoked by dots with a radius of 2.30 deg (SD 0.17) that moved at 63.8 deg/s (SD 3.1).

Figure 3c depicts the effects of dot size and speed on OLR gain. The best model for these data was poly23 (R^2^ = 0.96; poly22 vs poly23: F(3,11) = 12.2, p < 10^−4^; poly23 vs poly33: F(1,10) = 1.60, p = 0.23). This analysis revealed that the mice most accurately followed motion stimuli with a dot radius of 2.96 deg (SD 0.31) and a speed of 31.9 deg/s (SD 2.89), reaching a gain of 0.72 (SD 0.04).

**Figure 4:**
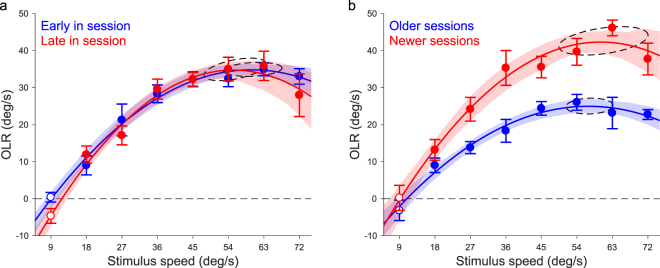
Consistency of technique Re-analysis of the data of the first experiment (Fig. 1). (**a**) OLRs observed in the first 20 minutes (blue) of sessions were not significantly different from those observed in the final 20 minutes (red). (**b**) OLRs observed in the later half of the sessions (red) were significantly larger than those in the first half (blue). Error bars represent SEM. Shaded bounds represent 95%-CI of the bootstrapped fits. Dashed ellipses represent 95%-CI of the peak estimates of those fits. Open markers indicate no significant difference from 0 (t-test, p > 0.05).

Finally, we examined the consistency of the behavioural measurements. We did this by re-analysing the data of Experiment 1 (Effect of speed; Fig. 1) in two ways. First, we compared OLRs measured in the first half of the 40-minute sessions to those observed in the second half (Fig. 4a). There was no difference between the OLRs of the early and late halves of the session as is apparent from the lack of separation of the 95%-CI boundaries of these curves. This may be contrary to predictions based on physical fatigue or neural habituation to visual motion. Next, we compared sessions performed before the middle date of each mouse’s test period (Fig. 4b) to those performed on or after that date. We found that the OLR was almost twice as strong in the newer sessions than in the older ones. This is contrary to our prediction that, by removing the visual effect from the OLR by head-fixing the mice, the reflex would attenuate over time. However, the estimates of the speed that evoked the strongest OLR did not change, that is, the 95%-CI ellipses of these estimates overlap on the stimulus speed axis.

## Discussion

The opto-locomotor reflex method presented here to measure mouse visual function is closely related to other methods monitoring the reflexes of eye- and head movements to onsets of large moving patterns. In the literature this is often referred to as the optomotor, optokinetic (OKR), or vestibulo-ocular reflex (VOR)^51^. In those methods as in ours, the animals perceive a sensation of self-motion induced by moving patterns that cover a large part of the visual field. The mice will try to compensate for this by turning in the same direction as the stimulus motion.

An advantage of our method is that the technique relies on voluntary running behaviour of the mice on the treadmill. The OLR is recorded automatically, removing the need of manually scoring the head movements, which is laborious and may be prone to subjective biases, although automatic video-tracking based systems have recently been introduced that do not have those disadvantages^49^. A main disadvantage of our technique compared to other OKR and VOR paradigms is that our method requires a surgery to attach the head post. This surgery, however, is short and minimally invasive, and may be required anyway in case the mouse is also used for concurrent imaging or neurophysiological recordings. Our method could further be refined by the addition of eye tracking equipment to measure reflexive eye movements.

On average across the three experiments in this study, the mice exhibited the highest OLR-gain at a pattern speed of 32.6 deg/s, which is faster than optimal speeds in the range of 12 deg/s that are mostly reported in other OKR or VOR studies^17,29,48,49,52–56^. This discrepancy may be due to the fact that all these studies use gratings instead of dot stimuli. A recent study with moving random dot patterns shows, in a two-alternative forced choice task, that mice are most sensitive for speeds in the range of 50 deg/s^57^. Another possible explanation for the discrepancy in optimal speeds is the fact that we use locomotion (running) as read-out as opposed to reflexes of the head or eyes measured in other studies^17,29,48,49,52–56^.

The results of the contrast experiment showed a slight decline of the OLR in response to the highest contrast value, whereas other studies reported a monotonic increase of sensitivity with contrast^29,44,58^. An explanation of this discrepancy may lie in the way that we manipulated the contrast of our stimuli. We used dots with luminance values ranging from 0.11 to 3.98 cd/m^2^ but kept the background at a constant 0.09 cd/m^2^. This means that not only the contrast changed, but also the overall luminance of the display. Perhaps at the highest level, the stimulus was so bright that it slightly dazzled the mice, reducing the effective contrast. The use of dark and bright dots that deviate equally from a mid-grey background should remove this confound.

We found that a dot radius of about 3 degrees evoked the highest OLR gain. Although the dot size and the spatial frequency of gratings cannot directly be compared, this diameter is on the same order of magnitude as the optimal period of about 10 degrees found for gratings in OKR and VOR experiments^35,59,60^, taking into account that the positive (bright) part of a 10-degree period is 5 degrees. By presenting the stimuli at a range of speeds, we are able to assess the interaction between speed and dot size. The contours in Fig. 3d exhibit a clear slant in the area of the plot where the dots were large and the stimulus speed low. This means that the parameters speed and dot size interacted, corresponding to the verbal description of the transition from inverted-U tuning to the linear dependence given in the Results section. Some slant is also apparent in Fig. 3b but to a lesser degree because OLRs at 18 deg/s, although by definition as close to linear as the corresponding OLR gains, are relatively small compared the OLRs induced by faster moving stimuli. A comparable interaction has been observed^54^ between the tuning to the spatial and temporal frequencies of gratings under similar photopic conditions, but not between spatial frequency and speed. Because the latter is most similar to our dot size and speed tuning, this raises the question why this interaction occurs in broad band stimuli but not gratings.

In summary, the method presented here provides a fully automated readout of mouse visual function that removes the need for behavioural training. It’s sufficiently sensitive to relatively subtle changes in the stimulus parameters and is consistent in at least the within session timescale. Moreover, it can be straightforwardly combined with research techniques that require the head to be fixed in space. We conclude that the OLR method is a useful addition to the mouse visual neuroscientific toolbox.

## Materials and Methods

### Animals

In this study, we used 6 male wildtype C57BL/6 J mice. We chose this strain because it possesses normal vision^35,36,60–62^ and is most often used in mouse perceptual/behavioural studies. Habituation of the animals began by handling and hand-feeding the mice each day. After one week, when the animals were at ease around the experimenter, we determined their starting weights (24.8 ± 1.2 g). We gave each animal 2.2 g of food per day, and each day we checked that they were between 85% and 95% of their starting weight. If animals fell below the 85% (above the 95%) threshold we increased (decreased) feed. All experiments were conducted in compliance with Dutch and European laws and regulations and were approved by the animal ethical committee of Radboud University Nijmegen.

### Surgery

Anesthesia was induced and maintained by isoflurane (4% at induction, 1.0–1.5% maintenance). The anesthetised animals were placed in a stereotactic holder fixating their heads, with their eyes covered in sterile ocular lube (Puralube, Dechra) to prevent eye dehydration. After shaving the head of the animal, the skin on top of the head was removed, and a local anesthetic (1 mg/ml Lidocaine HCL with 0.25 mg/ml Bupivacain Actavis), was applied to the exposed periosteum and the skull was cleaned with a bone scraper. Finally, a custom-made titanium head plate was fixed to the skull with dental cement (SuperBond C&B, Sun Medical).

### Habituation

A week after surgery, the mice were handled and head-fixed in the set-up for ten minutes. The duration was then increased to 40 minutes per session over the following 5 days to habituate animals to prolonged head fixation on the treadmill. After 8–10 sessions of habituation, mice would run on the ball for 2 sessions of 40 minutes each per day. The animals were running spontaneously, without requiring rewards or prompting otherwise.

### Visual stimulation

**Figure 5:**
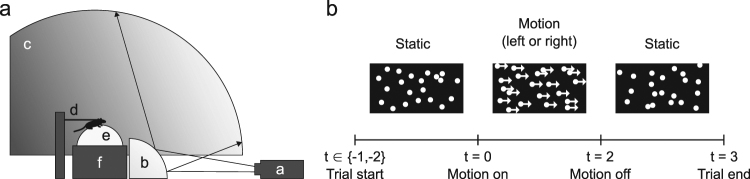
Experimental set-up. (**a**) Schematic drawing of the set-up. A projector (**a**) displayed patterns of randomly positioned dots via a mirror (**b**) onto the inside of a dome (**c**). Mice ran under head-fixed conditions (**d**) on a Styrofoam ball (**e**) floating on air (**f**). (**b**) Stimulus time course of one trial. Trials started with a static dot pattern. Motion onset (t = 0) occurred either 1 or 2 s after the start of the trial. The pattern of dots drifted either leftward or rightward for 2 s, producing optic flow consistent with leftward or rightward yaw of the mice, respectively. The trial ended with 1 s of static dots.

Stimuli consisted of light grey dots on a dark grey background. We used an Optoma X501 video projector (resolution: 1920 × 1080@60-Hz) to project the stimuli, via a quarter-spherical mirror, onto the inside of a dome made of fiberglass-reinforced resin (Fibresports UK; Basildon, UK) with an inner-diameter of 112 cm (Fig. 5a). We placed the mouse on a treadmill consisting of a Styrofoam ball floating on air in a custom-made socket (University College London workshops) at the center of this dome. The visual stimuli covered 220 degrees of visual angle horizontally, and from 10 degrees below the mouse to 80 degrees above it vertically.

The distribution of dots within this window was random and isotropic. More specifically, each dot was assigned an azimuth (in degrees) *azi* = R(−110°, 110°), where *R*(*a*, *b*) is a function that returns a random value from the uniform distribution over the interval (*a*, *b*), and an elevation (in degrees) *ele* = sind^−1^ *R*(sin d −10°, sind 80°), where sind^−1^ and sind are trigonometric functions that use degrees instead of radians. Taking the arcsine over the uniform interval serves the spherical isotropy of the dot-density. Without it, the density would increase at higher elevations because the closer to the zenith, the smaller the circumference of the horizontal cross-section of the dome.

The azimuth and elevation values were converted to regular (x, y)-pixel values for the projector by means of an inverse lookup table. We pre-created this lookup table by measuring the azimuth and elevation of 200 sample dots that we projected onto the inside of the dome in a 20 × 10-raster, evenly spaced in (x, y)-projector coordinates. When a dot needed to be displayed at an azimuth and elevation that fell in between these pre-measured values we obtained the corresponding Cartesian pixel coordinates with bi-linear interpolation.

To minimize distortion of the shape of the dots (that would occur where the light from the projector hits the surface of the dome at an oblique angle) each “dot” consisted of a cluster of 21 partially overlapping component-dots that were organized in three concentric circles around the central position. These component-dots were drawn as anti-aliased OpenGL dot primitives with a 5-pixel diameter, large enough to form a blob that was free of gaps. The azimuth and elevation coordinates of each component-dot were fed through the (*azi*, *ele*)-to-(*x*, *y*) lookup table. This way, the dots looked maximally circular from the perspective of the mice. In the remainder, when we write “dot” we mean such a cluster of component-dots.

At the beginning of each trial, a new set of static dots appeared at random positions in the stimulus window. After 1 or 2 s, these dots started moving for 2 s until they suddenly stopped and remained on the screen for another second (Fig. 5b). The 1 or 2-s duration of the initial static phase were randomly interleaved to reduce the animal’s ability to anticipate motion onset. We created motion by incrementing (or decrementing) the azimuths of the dots at each 17-ms video frame, resulting in a rotation around the vertical axis, or yaw.

We showed motion at speeds ranging from 0 to 72 deg/s in steps of 9 deg/s. The dot-luminances were 0.11, 0.20, 0.5, 1.66, and 3.98 cd/m^2^ on a background of 0.09 cd/m^2^, resulting in Michelson contrasts of 0.09, 0.37, 0.68, 0.89 and 0.95. Dots had a radius of 0.6, 0.9, 1.4, 2.3, or 3.7 degrees. The number of dots was inversely proportional to their surface area such that the portion of the display that was covered with dots was kept at 27% in all conditions. This way, the mean luminance of the visual field remained constant when we manipulated the dot size.

### Behavioural paradigm

The mice performed twice daily in sessions of approximately 40 minutes. To test the effect of stimulus speed, a session consisted of 10 repeats of a randomly interleaved set of 36 conditions (9 speeds, 2 directions, 2 starting times). The dot size and dot contrast sessions featured 5 repeats of 100 conditions (5 speeds, 2 directions, 2 starting times, 5 contrasts or sizes). Speed test sessions were repeated 7 times; the contrast and dot size tests 14 times. Hence, each condition was repeated 70 times.

### Recording and data analysis

The 2D treadmill consisted of a Styrofoam ball (19.7 cm diameter) that was floating on pressurised air in a semi-spherical socket^63^, adapted from insect studies^64–66^. We used an optical computer mouse to register the yaw in deg/s of the ball with a sampling rate of 60 Hz. Yaw is a proxy for OLR because it is the axis of rotation of the visual stimulus. First, we smoothed the yaw time series using a 100-ms boxcar filter. Then, we calculated for each trial the mean yaw during the final 500 ms before motion onset and subtracted this baseline from the yaw time series. Further data analysis steps are described in the Results section.

### Data availability statement

The datasets generated during and/or analysed during the current study are available in the Donders Repository, https://WebDAV.data.donders.ru.nl/dcn/DAC_62001435_01_902/.
